# Hydrodebridement of wounds: effectiveness in reducing wound bacterial contamination and potential for air bacterial contamination

**DOI:** 10.1186/1757-1146-2-13

**Published:** 2009-05-08

**Authors:** Frank L Bowling, Daryl S Stickings, Valerie Edwards-Jones, David G Armstrong, Andrew JM Boulton

**Affiliations:** 1 Department of Medicine Manchester Royal Infirmary, University of Manchester, Manchester, UK; 2 Manchester Foot Clinic, Manchester Community Health, Manchester, UK; 3 Department of Clinical Microbiology, Manchester Metropolitan University, Manchester, UK; 4 Department of Surgery, University of Arizona College of Medicine, Tucson, AZ, USA

## Abstract

**Background:**

The purpose of this study was to assess the level of air contamination with bacteria after surgical hydrodebridement and to determine the effectiveness of hydro surgery on bacterial reduction of a simulated infected wound.

**Methods:**

Four porcine samples were scored then infected with a broth culture containing a variety of organisms and incubated at 37°C for 24 hours. The infected samples were then debrided with the hydro surgery tool (Versajet, Smith and Nephew, Largo, Florida, USA). Samples were taken for microbiology, histology and scanning electron microscopy pre-infection, post infection and post debridement. Air bacterial contamination was evaluated before, during and after debridement by using active and passive methods; for active sampling the SAS-Super 90 air sampler was used, for passive sampling settle plates were located at set distances around the clinic room.

**Results:**

There was no statistically significant reduction in bacterial contamination of the porcine samples post hydrodebridement. Analysis of the passive sampling showed a significant (*p *< 0.001) increase in microbial counts post hydrodebridement. Levels ranging from 950 colony forming units per meter cubed (CFUs/m^3^) to 16780 CFUs/m^3 ^were observed with active sampling of the air whilst using hydro surgery equipment compared with a basal count of 582 CFUs/m^3^. During removal of the wound dressing, a significant increase was observed relative to basal counts (*p *< 0.05). Microbial load of the air samples was still significantly raised 1 hour post-therapy.

**Conclusion:**

The results suggest a significant increase in bacterial air contamination both by active sampling and passive sampling. We believe that action might be taken to mitigate fallout in the settings in which this technique is used.

## Background

Treatment of chronic wounds frequently requires a combination of medical and surgical therapy to effect successful healing. Non-viable tissue may serve as a source of infection and thereby retard wound healing or increase the risk for complications [1-3]. Therefore by removing necrotic tissue and reducing the bacterial load on the wound surface, wound debridement may assist in healing [4,5].

There are numerous wound debridement techniques available to the clinician [6]. Surgical (also known as sharp) debridement using a scalpel or a biopsy is considered the optimal method for rapidly cleaning the ulcer and converting it to an acute wound; however it can be painful and not all practitioners are trained or permitted to perform such procedures. Other mechanical forms of "sharp" debridement include pulsed lavage, ultrasound disruption of debris, and high-pressure water jet dissection of the wound surface [7]. These alternative techniques may possibly serve to reduce biofilm prevalence and local bacterial burden thereby stimulating the repair process. Additionally, they may be better able to debride superficial slough than traditional biopsy or scalpels. Over the past several years, these alternate mechanical methods of debridement have become increasingly commonplace both in operating rooms and in clinical settings worldwide and are generally well regarded by clinicians that employ them. We are, however, unaware of prior reports that have evaluated the potential for aerosolization of particulates, namely bacteria, into the peri-operative environment whist using these modalities.

The purpose of this study was therefore to evaluate the potential for aerosolization of microbes during hydrodebridement therapy and additionally determine the effectiveness of hydro surgery in reducing the amount of bacteria in a simulated infected wound.

## Method

Four porcine joints with skin intact were purchased on day 1 of the study and disinfected with 90% alcohol. Artificial wounds were created with a scalpel blade to produce three wound sites per specimen; a superficial wound (site 1), a deep wound without a sinus (site 2) and a deep wound with a sinus (site 3).

### Baseline sampling

Biopsies were taken from site 1 of each porcine specimen using a 6 mm sterile cutter. Samples were collected in normal saline for histology and scanning electron microscopy (SEM). Three swabs were taken from each site of each specimen.

Swabs were immersed in 1 ml of phosphate buffered saline (PBS) and vortex mixed to promote equal bacterial suspension. 0.1 ml of suspension was removed and added to 9.9 ml of PBS to produce a 10'2 dilution. Culture plates were inoculated with 50 ul using a Spiral Plater. Mannitol salt agar was used for detection and enumeration of *Staphylococcus aureus*, Nutrient Agar for *Pseudomonas aeruginosa *and MacConkey Agar for *Escherichia coli*. Plates were incubated for 24 hours at 37 degrees centigrade after which further dilution was achieved by adding 0.1 ml of the incubated sample to 9.9 ml PBS for 10'4 dilution. Plates were again inoculated and incubated as above. The resulting CFUs were counted using an image analyser.

Biopsies taken for microbiology were placed in 1 ml of PBS and weighed then vortex mixed. The contents were ground in a sterile grinder until the tissue was evenly homogenised then transferred to a sterile universal container for five minutes of further vortex mixing. This was then processed as for the swabs.

Biopsies for electron microscopy were fixed in 10% neutral buffered formalin for 48 hours to kill any bacteria. The formalin was removed by washing in distilled water then passing through various concentrations of alcohol to remove residual water before allowing drying. Gold was used as a sputter coating before being mounted for viewing on the SEM.

### Specimen inoculation

On completion of baseline sampling the artificial wounds on each of the four specimens were inoculated with various pathogens. Specimen 1 was infected with Oxford *Staphylococcus aureus*, specimen 2 with *Pseudomonas aeruginosa*, specimen 3 with *Escherichia coli*. Specimen 4 was infected with 1 ml of an overnight polymicrobial broth culture derived from a patient with a Methicillin-resistant *Staphylococcus aureus *colonised wound. The specimens were then incubated in a sterile container overnight at 37°c. Swab and biopsy samples were taken after incubation and again following debridement using the same method described for baseline sampling.

### Hydrodebridement

All specimens were debrided consecutively on the same day and in the same treatment room. The room was approximately 3 metres by 5 metres in size and representative of a typical outpatient clinic. In keeping with operating room requirements there was no controlled air flow. The clinical room was disinfected after each debridement and a two hour rest period followed when the proceeding treatment was a different specimen. This approach was in accordance with local infection control policy to allow for dispersal of any pathogens [8].

The Versajet operator had undergone training in the theory and practise of the Versajet in a clinical setting. During debridement the operator wore gloves, plastic apron, bonnet, visor and mask for protection against contamination and injury.

### Evaluation of air bacterial contamination: active sampling

Air sampling took place at three stages in the treatment process using the SAS-Super 90 air sampler (SAS). One hour before debridement the air was tested to provide baseline data. Specimens were presented for treatment with a dressing over the artificial wound which was removed immediately prior to debridement using a sterile non touch technique during which another air sample was taken. This was representative of clinical treatment sessions allowing for the possibility of aerosolisation of bacteria from the wound.

Specimens were debrided until "surgically" clean which took approximately five minutes to complete for the three sites on each specimen. During the procedure the air was sampled at 100 litres per minute on the right hand side of the Versajet operator. The SAS was positioned 2.5 metres from the operator at head height. Further 1 minute samples were collected following treatment completion after 5, 15, 30 and 60 minutes. All samples were analysed for microbial content.

### Evaluation of air bacterial content: passive sampling

**Figure 1:**
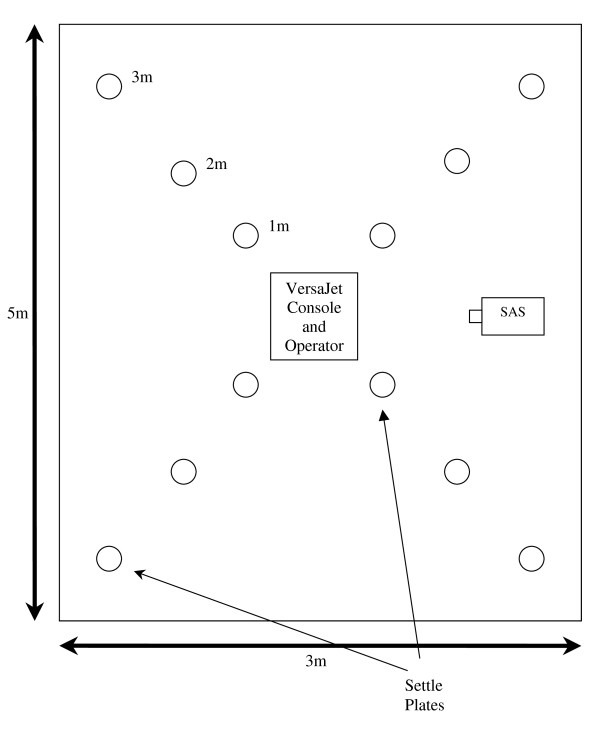
**Schematic diagram (plan view) of the treatment room layout illustrating position of Versajet, settle plates and SAS-Super 90 air sampler (not to scale)**.

In order to sample the air by passive methods 12 settle plates were situated around the sampling area. Figure 1 is a schematic diagram (plan view) of the treatment room layout illustrating the position of Versajet, settle plates and the SAS. Settle plates contained Tryptone soya agar (TSA) and were placed on the floor at 1, 2 and 3 metres from the active sampling area. Plates were 90 mm diameter. Settle plates were positioned 5 minutes after the removal of the dressing procedure, and then remained in situ for the 5 minute hydro surgery debridement and the subsequent 55 minutes. This made a total collection time for the settle plates of 1 hour. The settle plates were replaced prior to each new sample debridement.

Statistical analysis was by Minitab v15 (Minitab Inc., State College PA, USA). Significance testing on this parametric data was performed with t-tests.

## Results

### Microbiology

**Figure 2:**
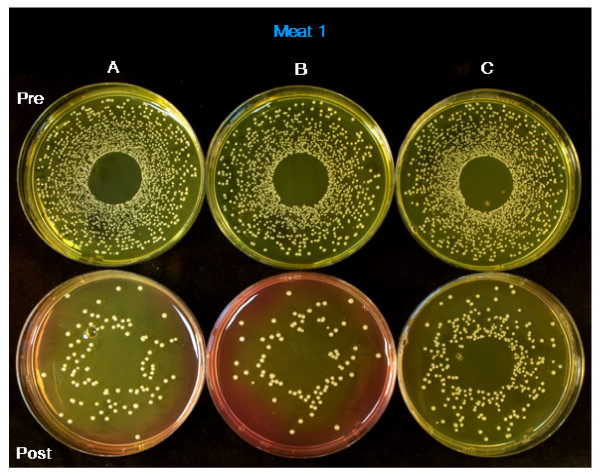
**This figure shows the *Staphylococcus aureus *count from specimen 1**. The top row shows pre-Versajet *Staphylococcus aureus *counts from the three sites and the lower row is the post Versajet.

No surface contaminating organisms were identified from the pre-inoculation sampling. Following debridement with the Versajet all wound sites in all specimens appeared clean and free from visible signs of infection. Bacterial counts obtained from specimens before and after Versajet treatment showed no significant difference. Five of the twelve swab samples (42%) showed a non-significant reduction in bacteria with a 1–1.5 log reduction in the post debridement bacterial count. The biopsy samples yielded up to 1 log reduction in bacterial counts with the *Escherichia coli *specimens showing the greatest decrease (Table 1). Wound type did not have an affect on bacterial numbers obtained pre and post treatment. Figure 2 illustrates *Staphylococcus aureus *counts from specimen 1 pre and post Versajet for all three wound sites.

**Table 1:** Mean bacterial count pre-versajet and post-versajet.

	Site 1	Site 2	Site 3	
	Swab CFUs/ml	Swab CFUs/ml	Swab CFUs/ml	Biopsy CFUs/ml
Specimen 1 (*Staphylococcus aureus*)				
Pre-Versajet	5.57 × 10^8^	2.57 × 10^8^	3.53 × 10^8^	4.43 × 10^7^
Post-Versajet	9.03 × 10^8^	7.97 × 10^6^	2.65 × 10^7^	2.13 × 10^6^
Specimen 2 (*Pseudomonas aeruginosa*)				
Pre-Versajet	5.54 × 10^7^	2.12 10^8^	7.68 × 10^7^	5.60 × 10^7^
Post-Versajet	5.38 × 10^7^	3.17 × 10^8^	3.07 × 10^8^	8.20 × 10^6^
Specimen 3 (*Escherichia coli*)				
Pre-Versajet	1.10 × 10^8^	1.57 × 10^8^	4.00 × 10^7^	3.00 × 10^7^
Post-Versajet	3.20 × 10^6^	6.47 × 10^6^	5.20 × 10^6^	5.67 × 10^6^
Specimen 4 *(Mixed wound organisms)*				
Pre-Versajet	1.17 × 10^7^	7.70 × 10^6^	4.57 × 10^6^	4.74 × 10^6^
Post-Versajet	N/A	2.43 × 10^6^	1.73 × 10^6^	4.07 × 10^6^

Note: site 1 = Small deep cut, site 2 = small deep cut with a sinus, site 3 = a superficial wound, N/A – not available.

### Results for active air sampling

**Figure 3:**
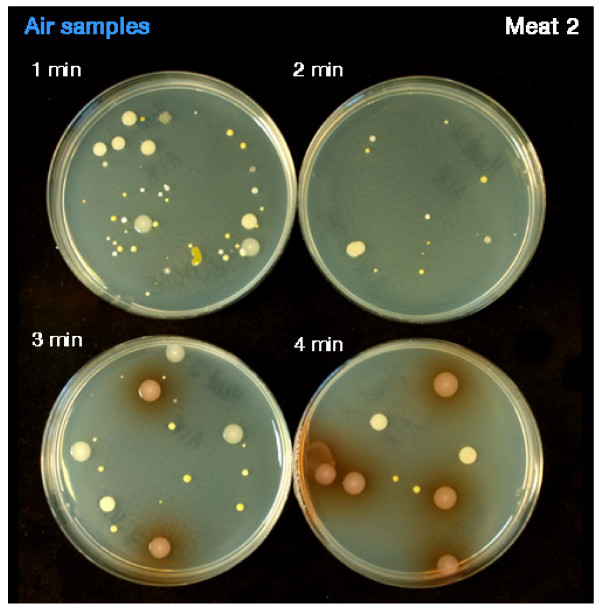
**Shows the air sampling during debridement using Versajet on specimen infected with *Pseudomonas aeruginosa***.

**Figure 4:**
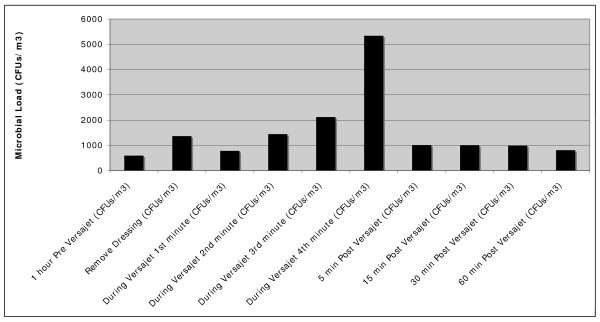
**The aerosolization effect of Versajet therapy pre, during and post Versajet debridement and dressing removal**.

During the actual debridement process the infecting organisms were isolated from the air and in the case of specimen 2 air samples contained *Staphylococcus aureus *from the previous debridement (Table 2). Figure 3 illustrates *Pseudomonas aeruginosa *isolated from active sampling during Versajet use. Microbial count levels ranged from 950 CFUs/m3 to 16780 CFUs/m3 during treatment. The mean bacterial counts for all samples per minute are shown in Table 3. Although counts decreased after treatment cessation the microbial load of air samples was still significantly raised one hour post therapy at 850 CFUs/m3 (Figure 4) compared to background levels. Air samples taken during dressing removal showed a significant increase (*p *< 0.05) in microbial counts relative to baseline (Table 3).

**Table 2:** Results of active sampling: number of colony forming units (CFUs) during each minute of the debridement process.

	CFUs/100 litres of air	Bacteria isolated
Specimen 1 *Staphylococcus aureus*		
I min	23	No SA
2 min	72	50 SA
3 min	TNTC****	SA +++
4 min	TNTC****	SA+++
Specimen 2 *Pseudomonas aeruginosa*		
I min	10	1 SA
2 min	36	5 SA
3 min	20	1 SA, 2 PAE
4 min	9	2 SA, 5 PAE
Specimen 3 *Escherichia coli*		
I min	9	
2 min	9	
3 min	15	
4 min	10	2 ECO
Specimen 4 *Mixed wound bacteria*		
I min	4	4 MRSA
2 min	0	
3 min	0	
4 min	7	

Note: **** = Versajet blocked SA = *Staphylococcus aureus*; PAE = *Pseudomonas aeruginosa*; ECO = *Escherichia coli*; MRSA = Methicillin-resistant *Staphylococcus aureus*; TNTC = To numerous to count.

**Table 3:** Results of active sampling: mean bacterial count by active sampling during dressing removal of each sample and for each minute of debridement of the four samples.

Sample	Remove Dressing (CFUs/m^3^)	Minute	During Versajet (CFUs/m^3^)
1	1415	1	775
2	1290	2	1440
3	1205	3	2115
4	1520	4	5335

### Results from passive air sampling

**Figure 5:**
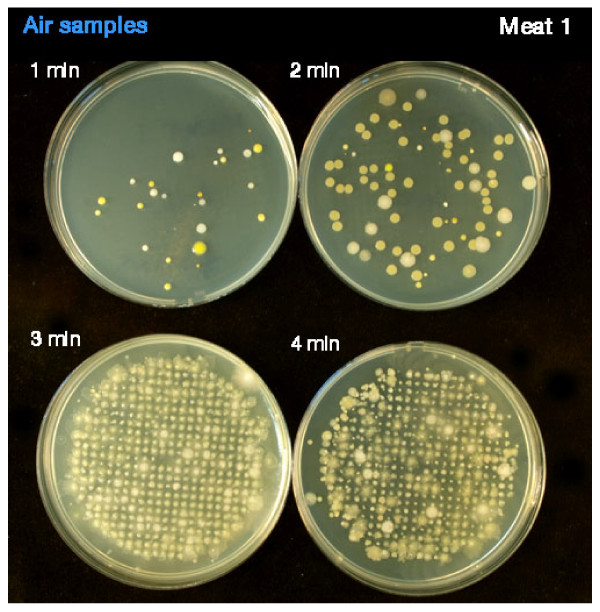
**Shows the air sampling during debridement using Versajet on speciemen infected with *Staphylococcus aureus***.

**Figure 6:**
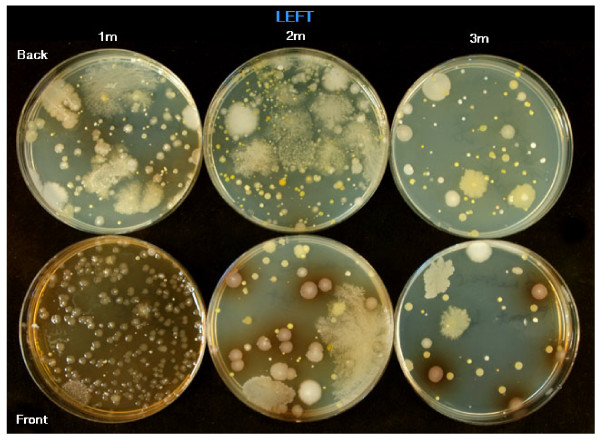
**Shows the settle plates whilst debriding using Versajet on the left side of the room (Front and Back) at 1 m, 2 m and 3 meters from the trolley**.

**Figure 7:**
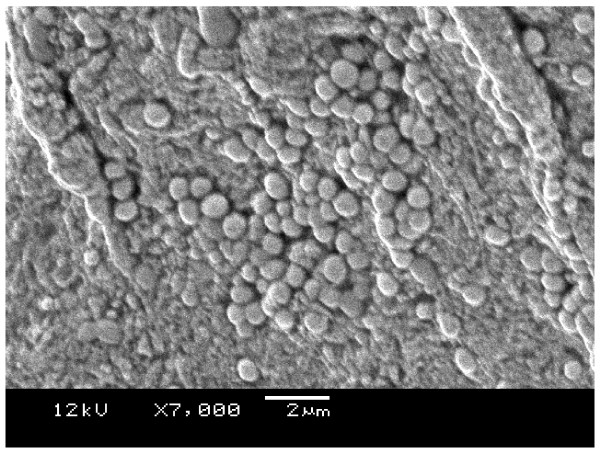
**Shows the adhesion of Methicillin-resistant *Staphylococcus aureus *on the sample after infection with wound bacteria × 7000 magnification**.

The results from the settle plates showed a higher number of CFUs in the 1 m and 2 m zones when compared to the 3 m zone (Table 4). During debridement of specimen 1 the Versajet became temporarily blocked and there was a large increase in CFUs on the settle plates to the extent that they were too numerous to count. This is shown in Figure 5. Figure 6 shows the settle plate bacteria from the left side of the peri-operative environment up to 3 metres away from the treatment trolley. The high number of CFUs is visible to the naked eye. Results from electron microscopy showed adhesion of bacteria to the specimen surfaces. Figure 7 illustrates Methicillin-resistant *Staphylococcus aureus*.

**Table 4:** Results from passive sampling: average bacterial counts at each settle plate location for all samples collected over a 1 hour period.

	Settle plates (back right)	Settle plates (front right)	Settle plates (back left)	Settle plates (front left)
1 m	85 ***	45	80	180**
2 m	46***	106**	80	47
3 m	44	0	72	32

Note: *** colonies include *Staphylococcus aureus *and Escherichia coli ** predominantly *Pseudomonas aeruginosa*

## Discussion

There is substantial empirical evidence that wound healing can be improved with surgical debridement and a general consensus among clinicians that debridement creates a favourable wound bed [4-6]. Positive outcomes reported include an increase in the percentage of granulation tissue and a marked decrease in slough.

One aim of this study was to examine bacterial load following hydro surgery debridement. No significant differences were found between bacterial counts of wound swabs or biopsies obtained pre and post hydro surgery independent of bacteria or wound type. Although wounds had an improved appearance after treatment there was no significant reduction in bacterial load.

However, it is clear from the CFUs illustrated in Figure 2 that hydro surgery can decrease the quantity of bacteria resident in a wound to some extent but not reaching significance. One wound site actually saw an increase in bacterial count post debridement. This occurred in a site designed to simulate a deep wound with a sinus. We suggest that this could be due to inaccuracies arising from the use of swabs to collect material from a deep seated, irregular and undermined wound with a sinus.

Despite not achieving statistical significance the results still demonstrate a decrease in the quantity of bacteria present in the wound but the question of where the organisms go needs to be addressed. Tables 2 and 4 provide evidence of aerosolization of bacteria both during and following debridement. Furthermore, this fallout appears to be displaced throughout much of the peri-operative environment as illustrated by Figure 6.

Of grave concern are the extreme bacterial quantities recorded when the debridement tool becomes blocked as seen in Figure 5. Our results showed irregular displacement of pathogens especially in the front right settle plates. The only area to avoid significant fallout was 3 meters in front and to the right of the clinician. It is not possible to account for equal or unequal fallout, due to the nature of high pressure spray but we can postulate that the SAS may have obscured the front right 3 meter settle plate.

During the active sampling process a count of 16780 CFUs/m^3 ^was obtained which is extremely high. A possible explanation would be that the CFUs visible during imaging had originated from more than one cell thus when the plate becomes crowded the actual number of visible CFUs does not represent a true figure. A statistical factor has been applied to allow for this hence a higher value is obtained.

Despite a two-hour time delay between debridement of different specimens cross contamination occurred (Table 2). The samples taken from specimen two, inoculated with *Pseudomonas aeruginosa*, also contained *Staphylococcus aureus *from the previous specimen.

Our results clearly demonstrate that there is a potentially high risk of contaminating the peri-operative environment during the process of hydrodebridement making cross infection a real possibility. Careful consideration of clinical location is necessary prior to using such debridement tools, particularly as hydro-surgery consoles are becoming increasingly used in community clinic settings globally. This is especially true in a climate where hospital acquired infections are under increasing scrutiny. The fallout recorded from dressing removal deserves the same consideration independent of hydro surgery and has implications for clinicians on a daily basis at every level.

The results from this study should not dissuade the clinician from utilising hydro surgery as an adjunct to other treatments but it is vital that action be taken to mitigate the bacterial fallout associated with its use. A transparent hood to cover the cutting tool and seal the affected area may reduce potential fallout, thus reducing bacterial contamination. Similarly, an improved cutting tool designed with bacterial fallout in mind could diminish contamination.

## Competing interests

The authors declare that they have no competing interests.

## Authors' contributions

FLB and VEJ conceived and designed the study. DSS and DGA conducted the statistical analysis. FLB and DSS compiled the data and drafted the manuscript and AJMB contributed to the drafting of the manuscript. All authors read and approved the final manuscript.
